# The Multitarget Activity of Natural Extracts on Cancer: Synergy and Xenohormesis

**DOI:** 10.3390/medicines6010006

**Published:** 2018-12-28

**Authors:** María Herranz-López, María Losada-Echeberría, Enrique Barrajón-Catalán

**Affiliations:** Instituto de Biología Molecular y Celular (IBMC) and Instituto de Investigación, Desarrollo e Innovación en Biotecnología Sanitaria de Elche (IDiBE), Universitas Miguel Hernández; 03202 Elche, Spain; mherranz@umh.es (M.H.-L.); mlosada@umh.es (M.L.-E.)

**Keywords:** cancer, natural compound, synergy, xenohormesis, polypharmacology

## Abstract

It is estimated that over 60% of the approved drugs and new drug developments for cancer and infectious diseases are from natural origin. The use of natural compounds as a potential source of antitumor agents has been deeply studied in many cancer models, both in vitro and in vivo. Most of the Western medicine studies are based on the use of highly selective pure compounds with strong specificity for their targets such as colchicine or taxol. Nevertheless, approximately 60% of fairly specific drugs in their initial research fail because of toxicity or ineffectiveness in late-stage preclinical studies. Moreover, cancer is a multifaceted disease that in most cases deserves a polypharmacological therapeutic approach. Complex plant-derived mixtures such as natural extracts are difficult to characterize and hardly exhibit high pharmacological potency. However, in some cases, these may provide an advantage due to their multitargeted mode of action and potential synergistic behavior. The polypharmacology approach appears to be a plausible explanation for the multigargeted mechanism of complex natural extracts on different proteins within the same signalling pathway and in several biochemical pathways at once. This review focuses on the different aspects of natural extracts in the context of anticancer activity drug development, with special attention to synergy studies and xenohormesis.

## 1. Introduction

Cancer is not a single disease, but a complex clinical situation in which multiple molecular pathways and cellular processes are compromised. Each cancer type has its own molecular fingerprint and, at least one of the cancer hallmarks described in [1], is altered. However, in spite of this heterogeneous situation, all cancers have a common behavior based on uncontrolled proliferation and invasion. This invasive phenotype is the real clinical problem and, in most cases, still remains unresolved, causing morbidity and mortality.

Anticancer research and drug discovery are continuously increasing the therapeutic arsenal, and relevant advances have been made towards individualized treatments [2,3,4,5]. New antibody-based drugs, called biological treatments [6,7], have improved treatments and prognosis in some cancer types such as breast, lung, liver cancers and lymphoma. New specific inhibitor families have also been developed, especially against proliferation related kinases. These drugs are called “inibs” [8,9]. In this sense, there is a tendency to look for highly specific drugs within the low micromolar range to solve very specific, almost individual cases. In vitro and in silico approaches have propelled the development of these specific drugs, and our bibliography is full of these examples [10,11,12,13]. These specific drugs follow the classic pharmacological dogma “one drug-one target”, but in spite of their undeniable value, they lack some relevant aspects that will be discussed in the following sections.

On the contrary, the molecular promiscuity of some molecules, especially those from natural origin [14,15], allow them to exert a potential multitarget mechanism of action. These compounds are able to interact with different targets, modifying different pathways or different steps of the same signaling cascade. In addition, promiscuity is not always due to a single compound but a mixture of compounds, as occurs in some complex natural extracts. In these cases, each compound is able to interact with one or multiple targets, increasing the pharmacological promiscuity of the whole drug. In addition, natural extracts or their main components can be combined with conventional chemotherapy, reducing the development of resistance to antitumor drugs and toxic effects [16]. 

This review will be focused on discussing the advantages and drawbacks of the use of natural extracts when compared with classical individual targeting strategy. Xenohormesis, multitargeting, synergy and drug resistance will be the main points that will be addressed.

## 2. Natural Compounds, Hormesis and Xenohormesis

Chemistry and analytical advances have allowed the synthesis and characterization of millions of new molecules for drug discovery. In some cases, synthesis was structurally guided using in silico or structural approaches, in others, combinatorial libraries were built based on a molecular scaffold or leads. This approach has allowed the development of new drugs not only for cancer treatment but also for other diseases. However, all these drugs have been human-designed and lack natural origin. On the contrary, natural extracts and their compounds have been selected for by millions of years of evolution as complex sources of medicinal agents. This is the basis of xenohormesis hypothesis [17,18]. They present very diverse chemical structures, from the simplest phenolic acids in plants to the most complex marine compounds [19,20,21]. Glycosylation, methylation and other esterifications and the presence of other moieties, increase the number and diversity of natural compounds. This constitutes a countless and invaluable source of new drugs. From a biomedical point of view, hormesis is an adaptive response in which the exposure to a low dose of an environmental factor or chemical compound, that is harmful at high concentrations, has a beneficial and/or adaptive effect on a cell or organism. Sometimes this response is mediated by some compounds that, when incorporated in the heterotroph diet, induce biological responses leading to pharmacological effects. This final effect is called xenohormesis, as the final benefit is obtained by the heterotroph organism, not for the plant that originally adapted to the stressful condition [17,18]. Xenohormesis is a way of cross-species interaction and communication. 

Although hormesis is an essential concept in evolution, xenohormesis has also allowed the expansion and fixation of evolutionary advances in non-autotrophs organisms. Nowadays, xenohormesis gives us a chance to obtain benefits from natural compounds and obtain new drugs selected by nature all through the evolution process. These compounds can be used directly for anticancer drug discovery, and also as new leads in novel developments using the classical structurally guided or in silico approaches. This is one of the main advantages of natural extracts and their compounds. They can be used as any other drugs, but with the benefit of being selected by natural evolution. 

## 3. Combined Therapies, Multitargeting and Synergy

Based on the classic pharmacological dogma “one drug-one target”, monotherapy has been the traditional approach, not only to treat diseases, but also to find new active drugs against a chosen target. However, presently there is evidence pointing out that combined therapies are much more efficient than single-drug-based treatments. In this sense, combinational therapy is extended to treat not only cancer but also other diseases, such as AIDS, bacterial infections, hypertension, metabolic or rheumatic disorders [22].

Combined drug therapy design is a hard and challenging task. Individual and combined actions must be characterized and it sometimes requires new preclinical and clinical trials. In addition, these multiple comparisons are sometimes difficult to incorporate into those studies. Combined therapies are normally based on co-administration of two or more drugs. These combined drugs can be based on the combination of pure compounds, or can be achieved by using drugs based on mixtures of natural extracts. 

Combined therapies are based on targeting different molecular signatures of the disease. On the one hand, multitargeting makes drug screening more complicated as complex assays must be developed to test all possibilities. On the other hand, multitargeting creates the opportunity to obtain synergic interactions between the combined therapy elements and drug resistance development, as detailed in further sections. There is a plethora of combined chemotherapy treatments covering most of the different cancer phenotypes. In fact, most of the recommended treatment regimens include two or more drugs, but as mentioned above, combined therapy use is not exclusive to cancer treatments, it is also extended to other disciplines such as antimicrobial and antihypertensive diseases.

Synergy is therefore the most relevant characteristic of combined therapies, including natural extracts. The first impetus for synergy research came from pharmaceutical legislation which demands the verification that every compound of a combined pharmaceutical preparation contributes to the claimed complete efficacy [22]. In terms of pharmacology, synergy is the ability of some mixtures to be more potent than the sum of their individual components. This definition is exportable to other disciplines and is based on the complementary as well as additive mechanism of action. Accordingly, synergy is not an absolute factor and the pharmacological interaction between the components of a mixture can be more or less synergic. This aspect is the main difference between additive and synergic behavior and is sometimes forgotten by researchers and clinicians. 

Another aspect to be analyzed is that a single mixture is able to perform in a different way depending on the proportion of its components. According to this, different proportions of the same compounds could provide different results in terms of synergy, not only in an absolute way, but also in terms of being more or less synergic [23]. In this sense, an ideal proportion which provides the highest synergy results is always mathematically possible.

**Figure 1:**
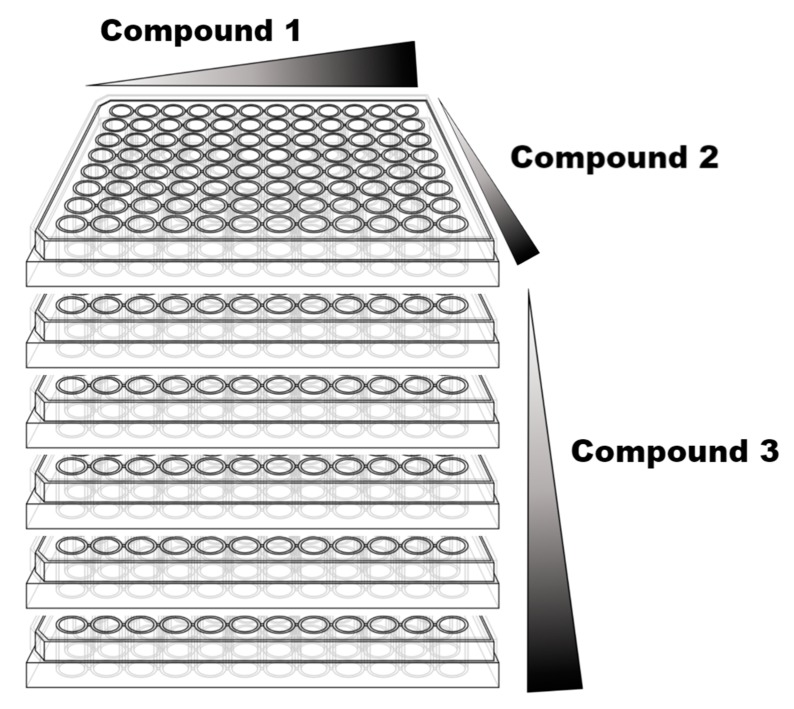
Checkboard plate design can be used not only for single plate experiments but also for more complex studies using three different compounds. In these cases, the concentration of each compound increases in one of the three dimensions (x, y and z axis) as indicated in the figure.

There are several ways to develop synergy studies, but in all cases, a previous and detailed design is required to obtain conclusive results. Most synergy studies fail because of a deficient design, both in qualitative and quantitative ways. There are several exhaustive and relevant reviews on synergy [22,24,25,26], so this review will not go deeper into how to study it. However, some aspects about study design deserve a comment:

- Drug selection: The right selection of the drugs for synergy studies is the first step to succeed. There are multiple available drugs for a single disease, but not all are suitable for a synergy study. Drugs must be selected considering different molecular targets. If not, antagonism or other undesired pharmacological interactions can be obtained. These different molecular targets can be located in different molecules, in distinguished epitopes of the same molecule or even in molecules of different pathways.
- Synergy study method: As mentioned above, there are several methods to study synergy between drugs. Quantitative methods such as Combination Index (CI) [27] or Fractional Inhibitory Concentration Index (FICI) [14] calculation are preferred as they obtain better conclusions.
- Biological assay: According to the selected method for synergy studies, a robust and reliable biological assay must be selected that allows testing a high number of samples with a large variability in composition. Survival or viability tests are diverse and allow high throughput screening approaches [28]. They are commonly used for anticancer compound research, but also for most of the other areas of drug discovery in which synergy is topical, such as antimicrobial drug discovery.
- Sample testing: Once the test is selected, an adequate design of the plates is also crucial. Checkboard plate design is probably the best approach for pairwise combinations using multi-wells plates. This strategy can be used not only for pairwise combinations but also for 3-drug combinations as shown in Figure 1.

Following these recommendations, the final results will not only be scientifically relevant, but also comparable to other single drug or combined therapies. This will allow researchers and clinicians to obtain better conclusions and contribute to the development of new therapeutic approaches.

## 4. Examples of Synergy Studies

References to synergic interactions between drugs in cancer research are abundant in the bibliography. However, focusing on natural extract synergy studies, three main groups of examples can be classified. The first group includes studies covering complex extracts whose components present synergistic interactions among them. The second group includes examples of synergy between different extracts and natural compounds of different origin. Finally, the third group comprises examples of anticancer approved drugs combined with natural compounds or extracts. Some of the most relevant examples of each category are listed below and shown in Table 1.

Pomegranate polyphenolic extracts have demonstrated numerous biological activities such as antioxidant, cardiovascular preventive and antitumoral activities [45,46,47,48]. These extracts are enriched in ellagitannins such as ellagic acid, punicalagin and punicalin. Synergy studies between pomegranate polyphenols have been performed in colon cancer cells [29], prostate [30] and other cellular models [49]. Grape fruit is also a well-known source of active compounds, including resveratrol as the most representative one. As with pomegranate, grape extracts present abundant biological activities [31,50]. In regards to cancer and synergy studies, grape polyphenols have shown synergy between them, especially on colon cancer cell models [32]. Rosemary terpenes have also shown antitumoral synergic activity on colon cancer cells [33]. Ginger compounds [34], graviola flavonoids and acetogenin’s action on prostate cancer [35] are examples of synergic interactions between different compounds included those that are present in the same extract.

In addition to single complex natural extracts, some studies have tested the synergic interactions between different extracts and/or different natural compounds, even mixing a complex natural extract with an individual natural compound. On the one hand, turmeric extracts have showed synergic activity with rosemary extracts as well as their representative compounds, carnosic acid and curcumin [36]. Tea natural compounds, mainly catechins, synergistically interact with capsicum compounds [37], soy phytochemicals [38,39] and between different tea extracts [40]. On the other hand, individual compounds such as resveratrol, quercetin and ellagic acid also interact synergistically in human leukemia cells [41,42]. Carotenoids and other phytochemicals also presented similar behavior [43]. Finally, natural compounds and extracts synergistically interact with clinically used anticancer drugs as occurs between many polyphenols and anticancer drugs such as cisplatin, doxorubicin, 5-fluoracil and others as reviewed in Reference [44].

## 5. Drawbacks of Using Natural Compounds

Pure natural compounds have demonstrated the same validity as synthetic or semisynthetic drugs in drug discovery. They are single compounds with a well-known chemical structure. They can be obtained from natural resources, in most cases using synthetic or semisynthetic approaches [51,52]. However, natural extracts, regardless of vegetal, microorganism or animal origin, are usually complex mixtures. Two main consequences are derived from natural extract’s complexity. First, these mixtures must be chemically characterized as much as possible. The improvement of analytical techniques such as liquid and gas chromatography coupled to mass detection, magnetic resonance and other approaches have permitted the characterization of very complex extracts [23,53,54,55]. The second consequence is that natural extract reproducibility is sometimes difficult due to the biological diversity of samples. This depends on origin, climate conditions, storage and extraction procedures. However, as occurred in other disciplines, this drawback can be avoided by controlling crop conditions, origin and extract production.

Natural compound bioavailability is also a drawback that deserves attention. Natural compounds have very different structures and, therefore, their bioavailability depend on the individual compound [56,57,58]. On the one hand, some natural compounds are quickly and fully absorbed, reaching the plasma in their native form so providing significant plasmatic concentrations. On the other hand, other natural compounds have scarce absorption, high metabolized rates and a fast excretion process. In all these cases, plasmatic concentration are low and biological activities are difficult to infer. Some strategies have been developed to improve natural compound bioavailability. Such as the use of different forms of encapsulation [59]; as nanoparticles [60,61], emulsion [62] or liposomes [63]. These approaches have been used especially to increase the solubility of highly hydrophobic compounds and extracts, and to improve the bioavailability of hydrophilic compounds with low stability or poor absorption [64,65]. However, in the end, natural compounds are not quite as different to other drugs that also present bioavailability limitations, and each case must be studied independently taking in account its solubility and the ADME processes. 

Drug resistance is probably the most important problem of cancer treatments. There are many drug resistance mechanisms [66,67] and no drug is free to develop a resistant phenotype. Once again, natural compounds and extracts are not different to other drugs, and resistance phenomena may also take place. Combined therapies minimize the risk of drug resistance, as tumor cells that develops resistance against one of the drugs may be affected by other drugs or compounds present in the same mixture. In this sense, natural pure compounds perform as any other drug and can be used in combination to reduce resistance, but natural extracts are mixtures that may act as a combined therapy itself, contributing to a decrease in drug-resistant phenotypes. 

## 6. Concluding Remarks

The current state of the art shows that combined therapies using natural extracts or combination of natural compounds and polypharmacology are quite promising. This is due to both the synergic interactions between their components and the reduction of drug resistant phenotype risk. Natural extracts are naturally occurring mixtures that have been selected by hormesis processes that can be beneficial to humans according to xenohormesis theory. They are indeed real combined therapies that, if well selected and characterized, can be used for new anticancer drugs development. In this sense, the design of high-quality synergy studies is crucial and a supplementary effort in this sense is worthy. 

But not everything may be considered an advantage when using natural extracts, they share some drawbacks with conventional anticancer drugs. Poor bioavailability and drug resistance mechanisms are as common among natural extracts as with conventional drugs. However, the polypharmacological properties of natural extracts makes resistance more difficult and, as occurred with other conventional drugs, bioavailability problems can be addressed using different approaches such as encapsulation. The most important disadvantage of natural extracts is their complexity and reproducibility, but as mentioned above, technical advances and quality controls during processing can overcome this problem.

In conclusion, natural extracts are a promising source of new anticancer drugs, but also suppose a challenging issue. As in the past, natural extracts will continue to be used as sources to develop new anticancer agents. These compounds have been selected on an evolutionary basis that may suppose an advantage for their performance.

Nevertheless, the same criteria for safety, efficacy and quality that are required for their synthetic counterparts must be expected. Strong efforts must be applied in extract characterization and in synergy studies. Despite these challenges, natural extracts are an irreplaceable source of new anticancer compounds that undoubtedly will allow the development of new anticancer therapies in the future.

## Figures and Tables

**Table 1:** Examples of synergic interactions among compounds or compounds and approved drugs in cancer research. Main examples included in this manuscript are shown in this table, organized in rows. First and second columns indicate the name of the components among which synergy is obtained. Third and fourth and fifth columns show the cellular models in which synergy studies were performed (including their origin), the main effect and the bibliographic reference.

Extract/Compound	Synergy	Experimental Model (Cell Line)	Effect	References
Pomegranate extract	Among their compounds	Oral cancer (KB, CAL27), colon cancer (HT-29, HCT116, SW480, SW620) and prostate cancer (RWPE-1, 22Rv1)	Antiproliferative, apoptotic and antioxidant	[29]
Pomegranate extract	Among their compounds	Prostate cancer (DU 145)	Antiproliferative, antimetastatic and phospholipase A2 (PLA2) inhibition	[30]
Grape extract	Among their compounds and with Ara-C and tazofurin	Leukemia (HL-60)	Antiproliferative and apoptotic	[31]
Grape extract	Among their compounds	Colon cancer (HCT116)	Antiproliferative and apoptotic	[32]
Rosemary extract	Among their compounds	Colon cancer (HT-29)	Antiproliferative	[33]
Ginger extract	Among their compounds	Prostate cancer (PC-3)	Antiproliferative	[34]
Graviola flavonoids	Among their compounds	Prostate cancer (PC-3)	Antiproliferative	[35]
Turmeric extract	With rosemary compounds	Breast cancer (MDA-MB-453, MDA-MB-468, and MCF7)	Antiproliferative, G1 cell cycle arrest	[36]
Tea extract	With capsicum compounds	Cervical cancer (HeLa) and breast cancer (4T1)	Antiproliferative	[37]
Tea extract	With soy compounds	Mice *in vivo* model	Metabolic effect	[38]
Tea extract	With soy compounds	Prostate cancer (LnCAP) xenotrasplants	Antiproliferative	[39]
Tea extract	With others tea extracts	Review	Antioxidant, antimicrobial and antitumoral	[40]
Resveratrol	With quercetin and ellagic acid	Leukemia (MOLT-4)	Antiproliferative, apoptosis and cell cycle arrest	[41,42]
Carothenoids	With other phytochemicals	Prostate cancer LNCaP , PC-3 and DU-145) and breast cancer (MCF-7)	Antiproliferative	[43]
Genistein	With cisplatin, 5-fluorouracil, arsenic trioxide, doxorubicin, gemcitabine camptothecine and hidroxi-camptothecine	Pancreatic cancer (BxPC-3 xenograft, COL-357 and L3.6pl) colon cancer (HT29), hepatic cancer (HepG2, Hep3B, SK-Hep-1, HEpG2 xenograft), cervical cancer (HeLa) ovarian cancer (OAW-42), bladder cancer (TCC-SUP) and lung cancer (ME-180pt, UMSCC-5)	Antiproliferative	[44]
Curcumin	With 5-fluorouracil, oxaliplatin, cisplatin, etoposide, camptothecine and doxorubicine	Colon cancer (HT-29), ovarian cancer (2008 and C13) and human and rat glioblastoma cell lines	Antiproliferative	[44]
(-)-epìgallocatechin-3-gallate	With doxorubicin, gemcitabine and cisplatin	Carcinoma doxorubicin resistant (KB-A-1 xenograft), cholangiocarcinoma (Mz-ChA-1 cell line and xenograft) and ovarian cancer (SKOC3, CAOV3 and C200)	Antiproliferative	[44]
Quercetin	With doxorubicin, cisplatin, arsenic trioxide and temozolomide	Neuroblastoma and Edwing’s sarcoma cell lines, laryngeal cancer (Hep2), leukemia (U937 and HL-60) and astrocytoma	Antiproliferative	[44]
Resveratrol	With Cisplatin and doxorubicin	Acute leukemia (ML-2/DX30, AML-2/DX100 and AML-2/DX300)	Antiproliferative	[44]
